# Role of Charge Regulation and Fluctuations in the Conformational and Mechanical Properties of Weak Flexible Polyelectrolytes

**DOI:** 10.3390/polym11121962

**Published:** 2019-11-29

**Authors:** Pablo M. Blanco, Sergio Madurga, Claudio F. Narambuena, Francesc Mas, Josep L. Garcés

**Affiliations:** 1 Physical Chemistry Unit, Materials Science and Physical Chemistry Department & Research Institute of Theoretical and Computational Chemistry (IQTCUB) of Barcelona University (UB), 08028 Barcelona, Catalonia, Spain; s.madurga@ub.edu; 2 Facultad Regional San Rafael, Universidad Tecnológica Nacional & Instituto de Física Aplicada (INFAP), Universidad Nacional de San Luis-CONICET, 5600 San Rafael, Argentina; claudionarambuena@hotmail.com; 3 Chemistry Department, Technical School of Agricultural Engineering & AGROTECNIO of Lleida University (UdL), 25003 Lleida, Catalonia, Spain; garcesjl22@yahoo.es

**Keywords:** polyelectrolytes, charge regulation, charge fluctuations, weak polyelectrolyte, annealed polyelectrolyte, Monte Carlo simulation, semi-grand canonical ensemble, binding equilibria, conformational equilibria, constant pH ensamble, stretching, scaling law

## Abstract

This work addresses the role of charge regulation (CR) and the associated fluctuations in the conformational and mechanical properties of weak polyelectrolytes. Due to CR, changes in the pH-value modifies the average macromolecular charge and conformational equilibria. A second effect is that, for a given average charge per site, fluctuations can alter the intensity of the interactions by means of correlation between binding sites. We investigate both effects by means of Monte Carlo simulations at constant pH-value, so that the charge is a fluctuating quantity. Once the average charge *per* site is available, we turn off the fluctuations by assigning the same average charge to every site. A constant charge MC simulation is then performed. We make use of a model which accounts for the main fundamental aspects of a linear flexible polyelectrolyte that is, proton binding, angle internal rotation, bond stretching and bending. Steric excluded volume and differentiated treatment for short-range and long-range interactions are also included. This model can be regarded as a kind of “minimal” in the sense that it contains a minimum number of parameters but still preserving the atomistic detail. It is shown that, if fluctuations are activated, *gauche* state bond probabilities increase and the persistence length decreases, so that the polymer becomes more folded. Macromolecular stretching is also analyzed in presence of CR (the charge depends on the applied force) and without CR (the charge is fixed to the value at zero force). The analysis of the low force scaling behavior concludes that Pincus exponent becomes pH-dependent. Both, with and without CR, a transition from 1/2 at high pH-values (phantom chain) to 3/5 at low pH-values (Pincus regime) is observed. Finally, the intermediate force stretching regime is investigated. It is found that CR induces a moderate influence in the force-extension curves and persistence length (which in this force regime becomes force-dependent). It is thus concluded that the effect of CR on the stretching curves is mainly due to the changes in the average charge at zero force. It is also found that, for the cases studied, the effect of steric excluded volume is almost irrelevant compared to electrostatic interactions.

## 1. Introduction

Charge regulation (CR) is defined as the capability of ionizable macromolecules, nano-particles and surfaces, to modify their ionization state as a response to external physico-chemical perturbations. In contrast to strong polyelectrolytes, such as DNA or RNA, whose phosphate groups are charged in very different environmental conditions, weak polyelectrolytes are specially sensitive to changes in the pH-value or the ionic strength, solvent composition, interactions with metal ions or other charged molecules. The paradigmatic mechanism of CR is the binding of small ions present in solution, and, in particular, acid-base equilibria, due to the ubiquitous presence of protons in aqueous solutions [1,2]. In a wide range of situations, the physicochemical behaviour of charged polymers cannot be understood without the presence of CR. A few examples would be the stability of colloidal systems [3], protein-surface [4,5], protein-protein [6,7] and protein-polyelectrolyte [8,9] interactions, nano-particle coating [10], supramolecular chemistry [11,12], ligand-receptor binding in biochemistry [13,14,15], drug delivery [16], protein folding [17], among many others.

Besides the ability to modulate the electric charge to external changes, there are two relevant aspects of CR which make the difference compared to systems with fixed charge. Firstly, although CR can also take place in rigid structures, weak polyelectrolytes usually are very flexible. That means that, due to the presence of electrostatic interactions between charged groups, the system tries to minimize the electrostatic repulsion (or maximize the attraction) by means of changes in the conformational structure. In the same way, modifications in the conformational structure affect the interactions between charged sites and thus their ionization state. Conformational and ionization degrees of freedom are thus highly coupled [18,19]. The natural mechanism for this fact is the rotation of the chemical bonds. It has been recently shown that one can even build effective rotational potentials which explicitly depend on the pH-value [20,21]. A natural conclusion of this fact is that the stretching properties of weak-polyelectrolytes should depend up to some extent on the ionization state of the macromolecule (i.e., the pH-value and the ionic strength) since the application of an external force modifies its conformational structure.

In the last two-decades, the development of single-molecule force spectroscopy has lead to a huge expansion of the field of mechano-chemistry [22]. Mechanically induced chemical reactions or conformational transitions have been recently described both in neutral and charged macromolecules [23,24,25,26,27,28,29]. The stretching of strong polyelectrolytes such as DNA and RNA, two strong polyelectrolytes, have also been the subject of a number of studies, which results to be extremely dependent on the valence and concentration of the counterions [30,31,32]. It has been also shown that self-avoiding electrostatic repulsion forces produce new elastic regimes and scaling behaviors [33,34,35]. In a recent paper, our group explored the possibility of induced charge regulation by means of the application of an external force to a weak polyelectrolyte [36]. Mechanical stretching leads to an increase in the distance between charged groups and significant changes in the degree of protonation are observed at certain pH and ionic-strength conditions. In the same way, changes in the pH-value affect the extension/force curves.

A second aspect involved in CR is that the charge is no longer a fixed quantity but a fluctuating one, which can lead to surprising effects. For instance, it is well known that, under certain conditions, charge fluctuations (CF) can produce attraction between two macromolecules with the same average charge. This phenomenon was firstly predicted in a classical work by Kirwood and Shumaker [37], who used statistical mechanics perturbation methods and since then it has been verified in a number of simulation and experimental studies on protein-ligand, protein-protein and protein-membrane interactions [2]. Simulation methods have proved to be specially useful in the quantification of this effect by turning on and off the CF [6]. However, the influence of CF in the conformational and mechanical properties of flexible weak polyelectrolytes has been so far hardly addressed. In most the studies aimed determining the conformational properties (end-to-end distance, radius of gyration, persistence length, etc.) the charge is taken as a constant, sometimes by considering the polymer as a string with constant charge density [38,39]. In a number of works, CF are taken explicitly into account by regarding the polymer as a set of punctual protonating sites linked by rigid or harmonic bonds, so that rotational conformations are not taken into account [40,41,42,43].

The present work addresses the role of charge regulation and the associated fluctuations in the conformational and mechanical properties of weak polyelectrolytes by means of Monte Carlo simulations at constant pH-value, so that the charge is a fluctuating quantity. Once the average charge *per* site is available, we turn off the fluctuations by assigning the same average charge to every site. A constant charge MC (ccMC) simulation is then performed. With this aim, we make use of a model, described in Section 2, which accounts for the main fundamental aspects of a linear flexible polyelectrolyte that is, proton binding, dihedral angle rotation, bond stretching and bending. Steric excluded volume and differentiated treatment of short range (SR) and long-range (LR) interactions are also included. This model can be regarded as a kind of “minimal” in the sense that it contains a minimum number of parameters but it still preserves the atomistic detail. In Section 3, the effect of CF in the conformational properties of a weak polyelectrolyte at zero force is analyzed. Section 4 focuses on the influence of CR and CF in the scaling behavior at low forces, that is, linear and Pincus regime. Finally, Section 5 is devoted to the effect of CR and CF in the extension-force curves.

## 2. Charge Regulation and Stretching of Weak Polyelectrolytes

### 2.1. Minimal Model of a Weak Flexible Polyelectrolyte

**Figure 1:**
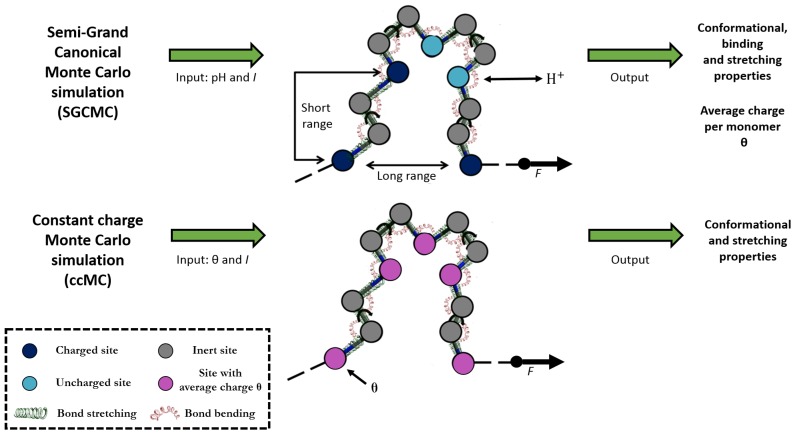
Outline of the two different kind of simulations of a weak flexible polyelectrolyte performed in this study: Semi-Grand Canonical Monte Carlo (SGCMC) and constant charge Monte Carlo (ccMC) simulations. In SGCMC, the pH-value is kept constant and the charge is free to fluctuate by means of proton equilibria (blue and cyan circles depict protonated and deprotonated sites, respectively). Conversely, in ccMC simulations, the charge of a site is fixed to its average value (purple circles). Grey circles represent inert sites. The bonds holding two ionizable sites are allowed to rotate. Bond stretching and angle bending and the mechanical stretching due to the action of an external force are also included in the model.

Let us consider a model, depicted in Figure 1, which captures the most relevant aspects of a weak flexible polyelectrolyte (proton binding, dihedral angle rotation, bond stretching and bending) but it involves a minimum number of parameters. Bond rotation represents the main mechanism of stretching at moderate and even relatively high forces. Bond stretching and bond angle bending are only relevant at very high forces (typically larger than 500 pN) for which the stretching regime is fully independent of the ionization state, as shown in a recent publication [36]. Assuming that the polymer is symmetric, (it presents planar symmetry when it is fully extended) complications due to tacticity can be avoided. As usual, the ionization state of the macromolecule is described by a set of variables $s=\left\{s_{i}\right\}$ with *i* = 1 ... *N*, where $s_{i}=1$ indicates that the site is protonated and $s_{i}=0$ otherwise. This is the basis of the Site Binding (SB) model [1]. The conformational state is determined by the set $c=\left\{\phi_{j}\right\}$, *j* = 1 ... *M*, where $\phi_{j}$ is the rotational angle of the bond *j*. It will be assumed that only the rotational states corresponding to energy minima (typically *trans* (*t*), *gauche*+ ($g^{+}$) and *gauche-* ($g^{-}$) ) are significantly populated. This is the central assumption of the Rotational Isomeric State (RIS) model, firstly proposed by Flory in order to calculate conformational properties of neutral chain molecules [44]. Combining RIS and SB models, we obtain the Site Binding Rotational Isomeric State (SBRIS) model, previously proposed in order to deal with ionization and conformational degrees of freedom on the same foot [18,19,21]. The SBRIS free energy can be expressed as

(1) $$F\left(s,c\right)=F_{\mathrm{rot}}+F_{p}+F_{E}+F_{\mathrm{lenght}}+F_{\mathrm{angle}}+F_{\mathrm{SEV}}+W$$

and

(2) $$\beta F_{\mathrm{rot}}=\sum_{j=1}^{M}\epsilon_{\mathrm{rot},j}\left(\phi_{j}\right)$$

epresents the RIS free energy contribution which is the sum of the torsional energies $\epsilon_{\mathrm{rot},j}\left(\phi_{j}\right)$ of the *M* rotating bonds and $\beta =1/k_{B}T$. Under the RIS approximation, we choose for $\phi_{j}$ the possible values $\phi_{j}=0$ (*t*), $\phi_{j}=+2\pi /3$ ($g^{+}$) and $\phi_{j}=-2\pi /3$ ($g^{-}$). Since the chain is symmetric, $\epsilon_{\mathrm{rot}}\left(+2\pi /3\right)=\epsilon_{\mathrm{rot}}\left(-2\pi /3\right)$. In this work, we will assume that the three rotational states have the same energy so that $\epsilon_{\mathrm{rot},j}\left(\phi_{j}\right)=0$.

The term $F_{p}$ represents the contribution of proton binding to the free energy

(3) $$\beta F_{p}=\sum_{i=1}^{N}\ln\left(10\right)\mu_{i}s_{i}=\sum_{i=1}^{N}\ln\left(10\right)\left(\mathrm{pH}-pK_{i}\right)s_{i}$$

where $\mu_{i}=\log\left(K_{i}a_{H}\right)$ is the reduced chemical potential corresponding to the proton activity $a_{H}$ and the protonation constant of site *i*, $K_{i}$. A site is positively charged when it is protonated. The chain contains a protonating site every three chain positions so that it can be regarded as a simplified version of linear poly-ethilene imine (LPEI), for which a complete conformational study is reported in Reference [19]. In the particular case of identical sites and bonds $pK_{i}=pK$.

The electrostatic interaction energy $F_{E}$ between charged sites is split into short range $F_{\mathrm{SR}}$ and long range $F_{\mathrm{LR}}$ contributions

$${F_{E}=F}_{\mathrm{SR}}+F_{\mathrm{LR}}.$$

This distinction is necessary due to the fundamental differences in the physical mechanism of SR and LR interactions. LR interactions are chemically unspecific and mediated by the solvent and they can be reasonably described by simple pair-potentials. In this work, the Debye-Hückel (DH) potential has been chosen

(4) $$\beta F_{\mathrm{LR}}=\sum_{i=1}^{N}\sum_{j=i+2}^{N}\frac{\ell_{B}}{d_{ij}}e^{-\kappa d_{ij}}s_{i}s_{j}$$

where $d_{ij}$ is the distance between the sites *i* and *j*, $\ell_{B}≃0.7$ nm and $\kappa^{-1}\left(\mathrm{nm}\right)=0.304/\sqrt{I\left(M\right)}$ represent, respectively, the Bjerrum and the Debye lengths in water at 298.15 K and ionic strength *I*. SR interactions between neighboring sites, however, are mediated by the macromolecular skeleton and they strongly depend on the chemical environment of the interacting sites [1,19]. As a consequence, they need specific parameters to be described. $F_{\mathrm{SR}}$ reads

(5) $$\beta F_{\mathrm{SR}}=\ln\left(10\right)\;\sum_{i=1}^{N-1}\epsilon_{\mathrm{int},3i-1}\left(\phi_{3i-1}\right)\;s_{i}s_{i+1}$$

where $\epsilon_{\mathrm{int},3i-1}\left(\phi_{3i-1}\right)$ corresponds to the interaction energy between two neighboring sites linked by a bond in a given rotational state $\phi_{3i-1}$. This term clearly couples the ionization and the conformational degrees of freedom, a characteristic feature of the SBRIS model.

The elasticity of the bond length $l_{j}$ and bond angle $\alpha_{j}$ are included *via* the harmonic potentials

(6) $$F_{\mathrm{length}}=\sum_{j=1}^{M}\frac{k_{\mathrm{length},j}}{2}\;{\left(l_{j}-l_{j,o}\right)}^{2},$$

and

(7) $$F_{\mathrm{angle}}=\sum_{j=1}^{M-1}\frac{k_{\mathrm{angle},j}}{2}\;{\left(\alpha_{j}-\alpha_{j,o}\right)}^{2},$$

where $k_{\mathrm{length},j}$ and $k_{\mathrm{angle},j}$ are the bond stretching and bending force constants and *$l_{j,o}$* and $\alpha_{j,o}$ denote the equilibrium length and the equilibrium bending angle of bond *j*, respectively. The stretching of the chain exerted by the the applied force F is quantified by the mechanical work

(8) W=−F r

where ***r*** is the end-to-end vector. Finally, $F_{\mathrm{SEV}}$ accounts for the steric excluded volume (SEV) effects, due to the finite size of the sites and chemical groups composing the chain. They are implemented by means of a hard-sphere potential

(9) $$F_{\mathrm{SEV}}=\sum_{i,j=i+4}^{M+1}\left\{\begin{array}{cc} \infty & d_{ij}\leq R_{i}+R_{j}\\ 0 & d_{ij}>R_{i}+R_{j} \end{array}\right.$$

where $R_{i}$ is the hard-sphere radius of site *i*. They will be turned on and off in the computations throughout this work in order to quantify their importance in the conformational and stretching properties. When SEV effects are switched off, all sites are considered to be a point charge, that is, $R_{i}=0\;\forall \;i$. More details about the model and its approximations can be found in Reference [36].

### 2.2. Constant Charge versus Constant-pH Simulations

In order to explore the effect of CR and CF on the structural and stretching properties of a single linear weak polyelectrolyte, we make use of a Semi-Grand Canonical Monte Carlo (SGCMC) code previously developed by our group [19,21]. This program has been recently used to study the possibility of inducing CR by means of mechanical stretching [36]. Since in the present work we are particularly interested in the behavior of the system when CR and CF are turned on and off, the code has been modified in order to perform both constant-pH (SGCMC) and constant charge Monte Carlo (ccMC) simulations, as outlined in Figure 1. In SGCMC simulations, the pH is the control variable and CR is explicitly taken into account. Statistical averages of the conformational, stretching and binding properties are computed and they are pH-dependent. The average charge per monomer θ is defined as

(10) $$\theta =\frac{<N_{+}>}{N}=\langle s_{i}\rangle ,$$

where *$<N_{+}>$* is the average number of protonated sites. Note that since the simulations are performed at constant pH, $N_{+}$ is a fluctuating quantity. The macroscopic quantity which measures the intensity of the fluctuations is the Binding Capacitance *C* [2,13] which is defined as the variance of the probability distribution of $N_{+}$

(11) $$C=\langle {\left(N_{+}-N\theta \right)}^{2}\rangle =N{\left(\frac{\partial \theta }{\partial \mu }\right)}_{F}$$

The second identity can be proved by means of elementary statistical mechanics. As commented in the introduction, CF are the responsible of interesting phenomena such as the effective attraction of macromolecules with the same charge sign, under certain conditions [37].

In order to assess the relevance of CF, we will compare the same conformational properties of interest obtained from ccMC and SGCMC simulations for the same average charge. Unlike SGCMC, in ccMC simulations CR and CF are switched off. The charge state of each site is imposed beforehand and kept constant at the value θ obtained from the equivalent SGCMC simulation, which means that θ is an output for SGCMC and an input for ccMC. Since correlations and CF are absent, the ionization and conformational degrees of freedom are now decoupled. The ionization states of two different sites become desconected. As a result, the average electrostatic interaction between sites *i* and *j* energy $\epsilon_{\mathrm{int},i,j}$ reads

(12) $$\langle \epsilon_{\mathrm{int},i,j}\left(c\right)s_{i}s_{j}\rangle ≃\epsilon_{\mathrm{int},i,j}\left(c\right)\langle s_{i}s_{j}\rangle ≃\epsilon_{\mathrm{int},i,j}\left(c\right)\theta^{2}$$

which is exact only in the limit of long enough distances between sites. This is equivalent to take $s_{i}=\theta$ in Equations (4) and (5). As a consequence, only conformational degrees of freedom are free to change and $F_{p}$ does not contribute to the free energy.

Concerning the conformational properties, special attention will be paid to the *gauche* state probability

(13) $$P\left(g\right)=\frac{\langle M_{g}\rangle }{M}$$

where $\langle M_{g}\rangle$ is the average number of bonds in the *gauche* state and to the persistence length $l_{p}$, defined as the average sum of the projections of all the bonds j≥i on a given bond *i* in an infinitely long chain

(14) $$l_{p}/l_{0}=\sum_{j\geq i}\langle b_{i}\cdot b_{j}\rangle$$

where $b_{i}$ denote unitary vectors pointing to the direction of the bonds. $l_{p}$ is related to the average square end-to-end distance $<r^{2}>$ by the relationship [44]

(15) $$l_{p}=\frac{<r^{2}>}{2Ml_{o}}+\frac{l_{0}}{2}=\frac{<{\left(r_{M+1}-r_{1}\right)}^{2}>}{2Ml_{o}}+\frac{l_{0}}{2}$$

where $<r_{i}>$ is the position of site *i*.

### 2.3. Parameters Used in the Simulations

We consider a chain with N=50 identical ionizable sites with pK=9, a large enough number to avoid end effects. The interaction parameters are $\varepsilon_{\mathrm{int}}\left(t\right)=1$ and $\varepsilon_{\mathrm{int}}\left(g^{+}\right)=\varepsilon_{\mathrm{int}}\left(g^{-}\right)=3$, which means that a bond holding two neighboring charged sites has a very small probability of being in the *gauche* state. As in LPEI, the chain consists of an ionisable site every two inert sites (i.e., 148 nodes or M=147 bonds), as shown in Figure 1. When excluded volume interactions are included, the sites are treated as hard spheres with radii R=1.7 Å and R=1.55 Å for inert and ionisable sites, respectively. These values were used in a previous study on the conformational and binding properties of LPEI [19]. For simplicity, all ccMC simulations have been performed without SEV effects, that is, with R=0. All bonds are considered to have equal bond stretching and bending parameters *$l_{o}=1.5$* Å, $\alpha_{o}=120^{\circ }$, $k_{\mathrm{length}}=300$ kcal ${\mathrm{mol}}^{-1}$ ${Å}^{-2}$ and $k_{\mathrm{angle}}=0.01$ kcal ${\mathrm{mol}}^{-1}$$\deg^{-2}$, which are the typical values used in Molecular Dynamics for C-C bonds [45]. Only the bonds with pending ionisable sites are allowed to exhibit free internal angle rotation ($\epsilon_{\sigma }\left(t\right)=\epsilon_{\sigma }\left(g^{+}\right)=\epsilon_{\sigma }\left(g^{-}\right)=0$) whereas the rest of the bonds are forced to remain in *trans* conformation. The simulations are performed at room temperature T=298.15 K. The reported results represent the average over 8 to 16 different SGCMC simulations, which have been equilibrated in the first $5\times 10^{7}$ configurations. The thermal averages have been computed in the following $1\times 10^{9}$ realizations. More computational details about the used algorithm can be found in the Supplementary Materials and explained in detail in Reference [36].

## 3. Effect of Charge Regulation in the Binding and Conformational Properties at Zero Force

**Figure 2:**
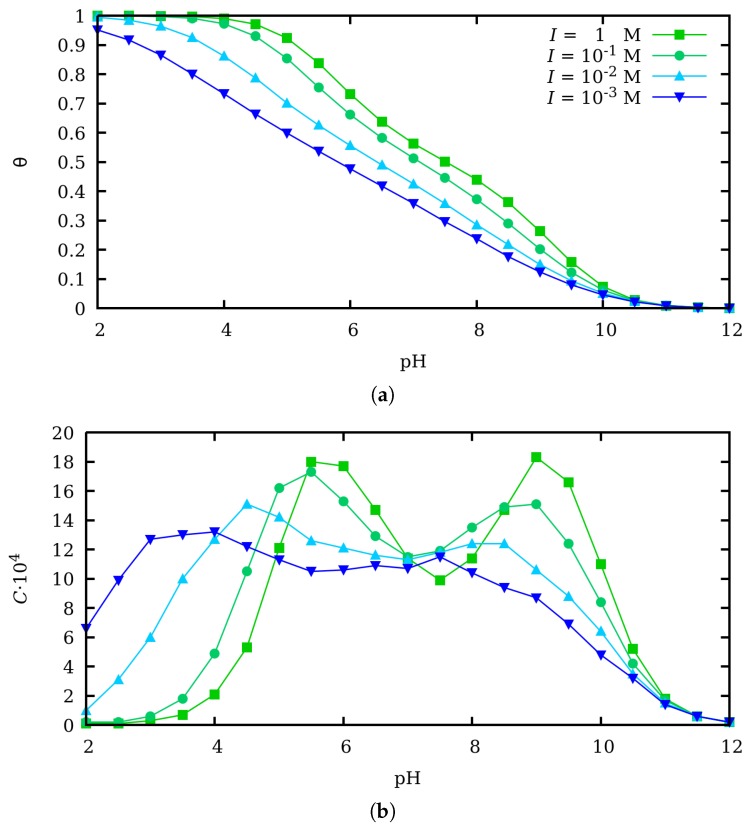
(**a**) Average degree of protonation θ and (**b**) binding capacitance *C* at ionic strengths (from top to bottom) 1 M (green squares), 0.1 M (turquoise circles), 0.01 M (cyan uppwards triangles) and 0.001 M (blue downwards triangles) obtained by means of pH-constant, SGCMC simulations. Steric excluded volume contribution is not shown because no effect is observed in the obtained θ-values.

Let us first analyze the influence of charge regulation on the conformational properties of the polyelectrolyte when no mechanical force is applied. Since the average degree of protonation θ is at once output from the SGCMC and input for ccMC simulations, we firstly discuss the dependence of this quantity on the pH-value. The resulting titration curves are shown in Figure 2a for four different ionic strengths ranging from 1 to 0.001 M (from top to bottom). As a general trend, it is observed that θ decreases when lowering the ionic strength, since electrostatic interactions become stronger and more energy is needed to protonate a site.

It is important to recall that in our model nearest neighbor (SR) interactions and LR interactions are treated in a different way. SR are described by chemically specific parameters ($\varepsilon_{\mathrm{int}}$) which, in accordance with experiments [1,19], are taken as independent of the ionic strength. At θ≃1/2, which corresponds to the plateau observed in the titration curve, an ordered structure consisting of alternated protonated and deprotonated sites is formed [46]. Due to the interaction with the two neighbors, the empty sites now bind protons with a different effective p*K*-value (roughly p$K-2\varepsilon_{\mathrm{int}}$). As a result, the titration curve resembles that of a system with two different p*K*-values.

Conversely to SR, LR interactions are described by the DH potential, which is strongly dependent on the ionic strength. LR interactions tend to destroy binding correlation between neighboring sites and it is more amenable to mean-field treatments. This is reflected in the shape of the titration curves when the ionic strength decreases, which becomes flatter and the plateau at θ≃1/2 progressively disappears. The differences between SR and LR interaction can be better understood by computing the binding capacitance *C* as a function of the pH-value, which is shown in Figure 2b. As expressed by Equation (11), *C* allows to directly quantify the intensity of charge fluctuations. It can be observed that, for large ionic strength, *C* presents two maxima at pH≃6 and pH≃9, which correspond to the inflection points in the titration curve and a maximum in the charge fluctuation. Furthermore, a minimum value is observed at pH≃7.5, related to the presence of the “ordered” alternating state at θ≃1/2. When the ionic strength decreases, *C* becomes progressively wider and flatter until the maxima and the minimum disappear as a result of the decorrelation introduced by LR interactions.

Let us turn CR and CF off and discuss the effect in the conformational properties. The θ-value obtained from SGCMC is now used as an input in the ccMC simulations, in which all the sites have a fixed charge equal to θ. Unfortunately, up to our knowledge, there is no established theory for the role of CF in the intra-molecular interactions and its consequences on the conformational structure of flexible weak polyelectrolytes. However, the contribution of CF in the force between two polyelectrolytes has been the object of a number of previous works, both from the theoretical and experimental point of view [2,6,37]. The interaction energy U(R) between two identical macromolecules with average charge *Q* separated by a distance *R* depend not only on their average charge but also on their binding capacitances. In absence of counterions, it is given by [2,37]

(16) $$\beta U\left(R\right)=\frac{\ell_{B}Q^{2}}{R}-\frac{\ell_{B}^{2}}{R^{2}}QC-\frac{\ell_{B}^{2}}{2R^{2}}C^{2}.$$

Probably, the most striking and counter-intuitive consequence of this theory is the presence of negative attractive terms in (16), despite the charge of both interacting molecules has the same sign. The first term corresponds to the usual coulombic repulsion. The second and third term in the r.h.s. of (16) are a direct consequence of CR and CF.

One of the objectives of this work is to clarify whether a similar effect can play a role, not only for inter-molecular interactions between charged macromolecules but in the intra-molecular interactions between different regions of a polyelectrolyte. If this was the case, one could expect CR to facilitate polymer folding due to the attractive contribution of CF. In order to put some light on this point, we have investigated the influence of CR in two quantities: the probability of a bond to be in *gauche* state, P(g), and the persistence length $l_{p}$. P(g) is a “local” conformational property, in the sense that it refers to the behavior of a single bond. The persistence length, $l_{p}$, however, is a “global” quantity, closely connected with the end-to-end distance given by Equation (15) and its value is the result of the behavior of many coupled bonds and sites.

**Figure 3:**
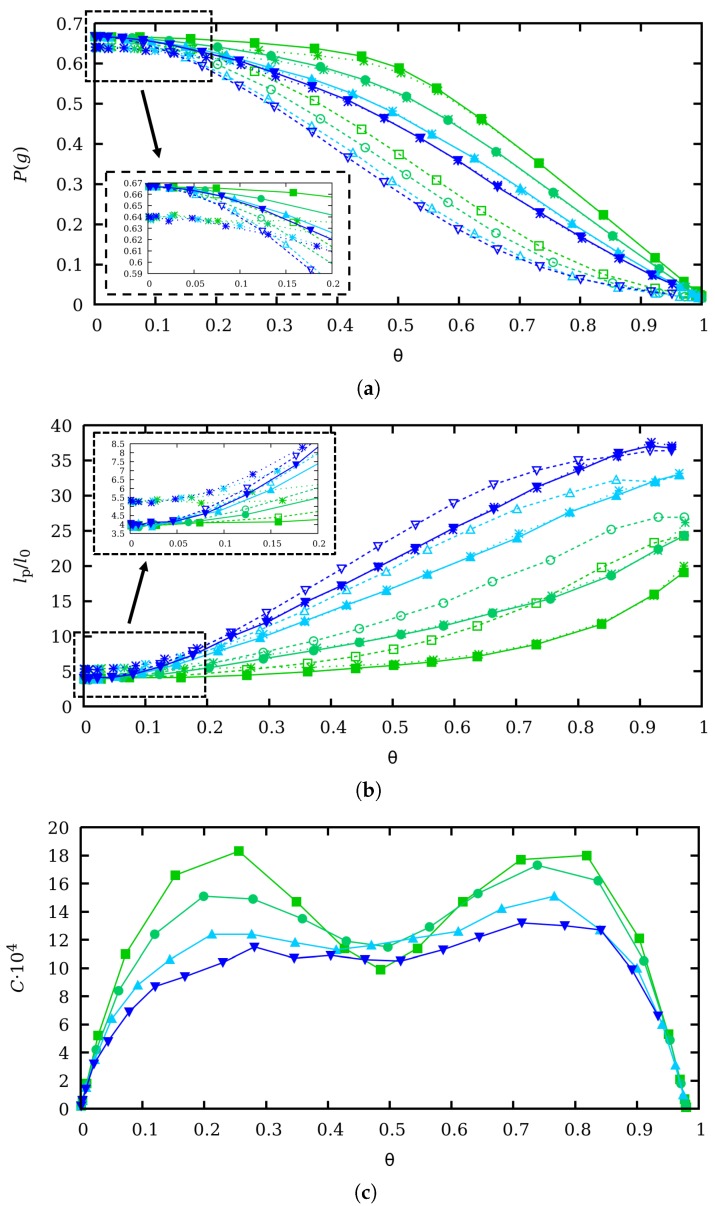
(**a**) *gauche* state probability, *P*(*g*), (**b**) persistence length $l_{P}$ and (**c**) binding capacitance *C* vs. the average degree of protonation θ at ionic strengths (from top to bottom) 1 M (green squares), 0.1 M (turquoise circles), 0.01 M (uppwards cyan triangles) and 0.001 M (downwards blue triangles). $l_{P}$ is normalized to the equilibrium bond length $l_{0}=1.5$ Å. Filled markers correspond to SGCMC without excluded volume, while empty markers correspond to ccMC simulations. In Figure 3a,b, star-shaped markers denote results obtained with SGCMC but they include steric excluded volume effects. The low charge regime is amplified in the insets of Figure 3a,b.

In Figure 3, the *gauche* state probability P(g) (a) and the persistence length $l_{p}$ (b) are plotted as a function of θ with CR (filled markers) and without CR (empty markers) for ionic strengths ranging from 1 to 0.001 M. We have also included the results of SGCMC simulations if the SEV effects are also present (star-shaped markers). Clearly, in all the studied cases, SEV effects are very weak and almost irrelevant compared to the self-avoiding electrostatic repulsions. The corresponding capacitance versus θ is also reported in Figure 3c. First of all, in Figure 3a it is clear that in both cases, with CR and without CR, P(g) decreases when the ionic strength decreases, since electrostatic screening is weaker, LR interactions are stronger and the macromolecule swells by forming more *trans* states. Moreover, two limiting behaviors for P(g) can be observed, which are also common to SMGMC and ccMC simulations. For low θ-values, the polyelectrolyte is uncharged so that P(g)→2/3 since the three conformational states have the same energy. When excluded volume is included, the asymptotic value is slightly smaller since some combinations involving *gauche* conformations are forbidden (See Figure 3a inset). For θ close to unity, the chain is fully extended in order to minimize the electrostatic repulsion, which implies that all the bonds are in *trans* state and P(g)→0. Clearly, there are no fluctuations in these two limiting situations and the binding capacitance is very small. However, at intermediate θ-values, CR and CF are important as the values of the binding capacitance indicate and clear differences arise between ccMC and SGCMC simulations. P(g) is significantly smaller without CR than with CR, independently of the θ-value and ionic strength. This fact can be explained because fluctuations allow to create uncharged regions in the chain which allow the chain to fold (through forming *gauche* states) while the extended regions preserve the total average charge. By assuming that all the sites have the same average charge, the possibility of the interplay between conformation and charge equilibria is lost and, as a result, the polyelectrolyte chain gets stiffer. This effect is confirmed by the behavior of the persistence length in Figure 3c. For intermediate θ-values (0.3<θ<0.8), ccMC clearly overestimates $l_{P}$ with specially significant deviations at high ionic strengths, that is, when CF are larger. As a conclusion, CR allows the chain to get more folded while the absence of CR makes it stiffer. Probably, a mechano-statistical theory based on first principles, similar to Kirkwood theory for interacting macromolecules [37], would be desirable in order to understand this point better.

## 4. Scaling Properties of Mechanical Stretching in the Low Force Regime

Let us discuss the role of charge fluctuations in the stretching properties at the low force regime, that is, when $F<k_{B}T/l_{P}≃1$ pN, under which the chain can be seen as a set of freely joined fragments with characteristic length equal to the Kuhn length $l_{K}$ [47]. This regime can be in turn divided into two different sub-regimes. For very low forces (F<0.3 pN), the extension $L_{z}$ responds linearly to force

(17) $$L_{z}/Ml_{0}=\beta F\frac{l_{K}}{3}=\beta F\frac{2l_{P}-l_{0}}{3}$$

which is a direct consequence of the fluctuation-dissipation theorem [48]. The Kuhn length $l_{K}$ is related to the persistence length as $l_{K}=2l_{P}-l_{0}$ [44]. It is important to note that $l_{P}$, as it will be shown in the next section, can only be considered constant in the low force regime. This fact is due to the activation of the rotational degrees of freedom at intermediate forces [36]. For forces ranging 0.3<F<1 pN, the action of electrostatic self-avoiding interactions makes the force/extension curve to follow the Pincus scaling law [33,48,49]

(18) $$L_{z}\propto F^{1/\nu -1}$$

which indicates the existence of a second low force sub-regime. The limiting value ν=3/5 corresponds to strong polyelectrolytes such as DNA whereas For ν=1/2 the linear behavior of a phanton chain is recovered.

**Figure 4:**
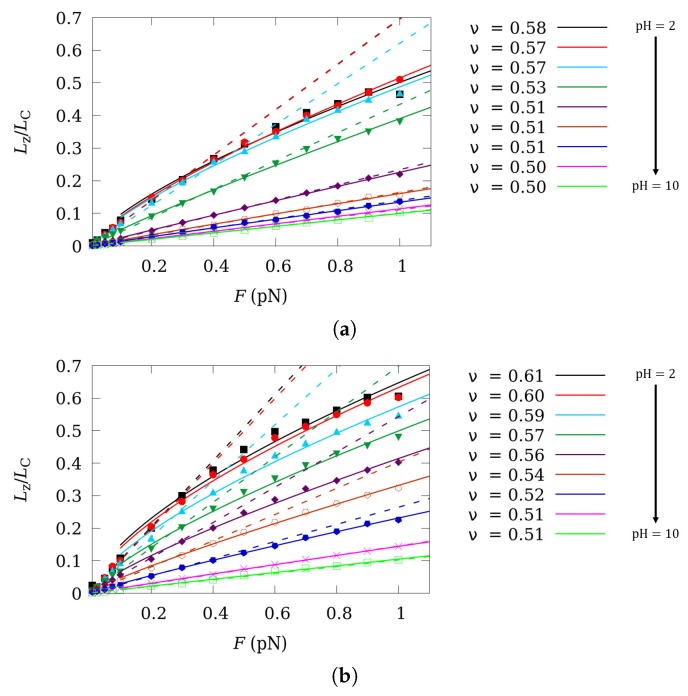
Normalized chain extension versus force curves in the low force regime for pH-values ranging from 2 to 10 (from top to bottom) obtained from SGCMC simulations without SEV at two ionic strengths 1 M (**a**) and 0.001 M (**b**). Dashed and continuous lines represent the best fit linear and Pincus scaling law, respectively. Chain extension is normalized to the contour length $L_{C}=Nl_{0}\cos\left(\left(\pi -\alpha_{0}\right)/2\right)$.

The extension/force curves resulting from SGCMC simulations (markers) are shown in Figure 4 for pH-values ranging from 2 to 10 (from top to bottom). Dashed lines are obtained solving Equation (17) using the $l_{K}$ value from simulations at zero force, at the corresponding pH and *I* values. The best fit of Pincus scaling law Equation (18) (continuous lines), in its range of validity 0.3<F<1 pN, is also plotted. Two ionic strengths are considered: I=1 M (Figure 4a) and I=0.001 M (Figure 4b). For large pH-values that is, when the polyelectrolyte is neutral, ν=1/2 and linear behavior is found for all the force-values. In the other limiting situation, for very low pH-values, when the polyelectrolyte is fully charged, simulations deviate from the linear behavior and they follow the Pincus scaling law (Equation (18)) with ν≃3/5, which nicely matches with the theoretical predictions [49]. The presence of SEV does not affect this conclusion, as shown in the Supplementary Materials.

**Figure 5:**
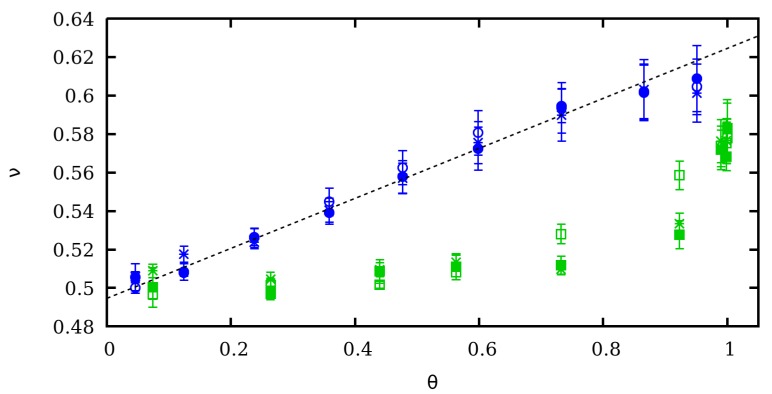
Pincus scaling exponent ν versus θ at two ionic strengths: 1 M (green markers) and 0.001 M (blue markers). The results of three different kind of simulations are plotted: SGCMC without SEV (filled markers), SGCMC with SEV (star-shaped markers) and ccMC (empty markers). Dashed and continuous lines represent the best fit linear and Pincus scaling law, respectively.

For intermediate pH-values and charges, a preliminary study [36] suggested that weak polyelectrolytes exhibit intermediate ν-values between the two limiting cases. However, whether such a transition is consequence of the CF or it is a direct consequence of the change in the average charge induced by modifying the pH-value is not clear. With this aim, we perform ccMC simulations using as input the average degree of protonation θ obtained from SGCMC at F=0 (Figure 2a). The scaling exponents ν resulting from SGCMC without SEV (filled markers), SGCMC with SEV (star-shaped markers) and ccMC simulations (empty markers) versus θ are reported in Figure 5 for two ionic strengths I=1 M (green squares) and I=0.001 M (blue circles).

Comparing the results obtained from SGCMC and ccMC simulations, it is observed that, in effect, ν presents a transition between linear and Pincus behavior both in presence or in absence of CR. Curiously, for I=0.001 M the transition between the two limiting ν-values is found to be linear with θ

(19) ν=mθ+n

where m=0.129±0.004 and n=0.494±0.001≃0.5 are fitted parameters to the SGCMC points. Best fitted Equation (19) is depicted as a black dashed line in Figure 5. The situation, however, becomes more complex for I=1 M, for which no significant deviation from the limiting value ν=1/2 is observed for θ<0.5. For θ>0.5. A drastic increase in the ν-value is formed when CR is taken into account. It is also worth mentioning that the limiting value ν=3/5 is not observed even at θ=1, when the polyelectrolyte is fully charged, independently of the presence of CF. This fact suggests that this could be also the case for strong polyelectrolytes at high ionic strengths. Again, SEV does not produce any significant effect in ν, even when the polyelectrolyte is almost uncharged (θ≃0).

## 5. Influence of Charge Fluctuations in the Intermediate Force Regime

**Figure 6:**
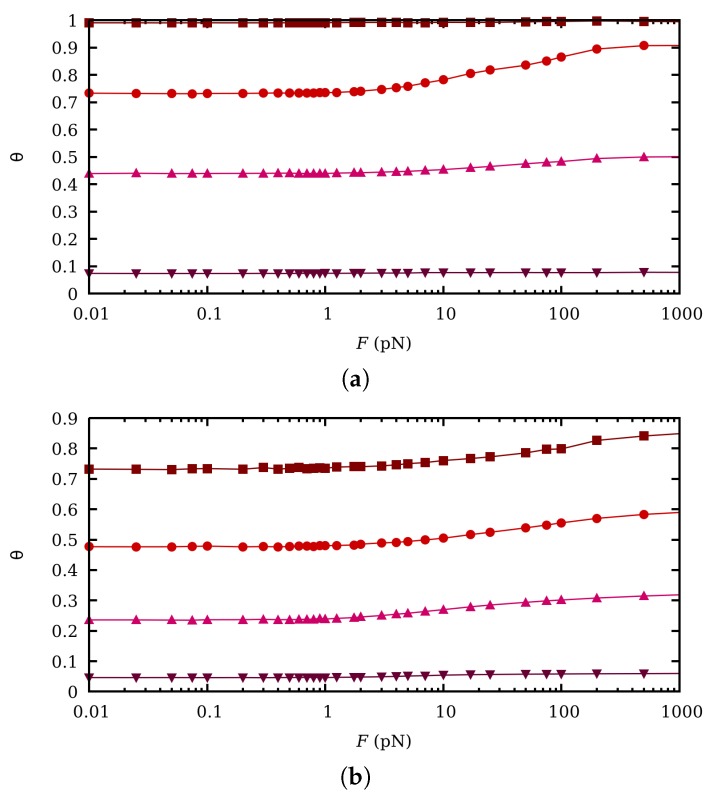
θ versus force curves at fixed pH-values 4 (wine squares), 6 (red circles), 8 (pink uppwards triangles) and 10 (purple downwards triangles), from top to bottom, and ionic strengths 1 M (**a**) and 0.001 M (**b**), the simulation results correspond to SGCMC without excluded volume.

It is well-established that conformational and binding degrees of freedom in weak polyelectrolytes are highly correlated because of charge regulation. Since in flexible polyelectrolytes, mechanical stretching dramatically changes the distance within the macromolecule, changes in θ are expected when an external force is applied. This effect has been extensively discussed in a recent publication [36]. θ versus force is plotted in Figure 6 at different pH-values (4, 6, 8 and 10) and for two ionic strengths I=1 M (Figure 6a) and I=0.001 M (Figure 6b). For intermediate pH-values and low ionic strengths, moderate CR is observed. This effect is significantly enhanced if the *gauche* bond state is favored, for instance, because of the formation of a hydrogen bond, although this issue is out of the scope of this work. Although this point would probably require a more intensive study, we present here some preliminary results.

**Figure 7:**
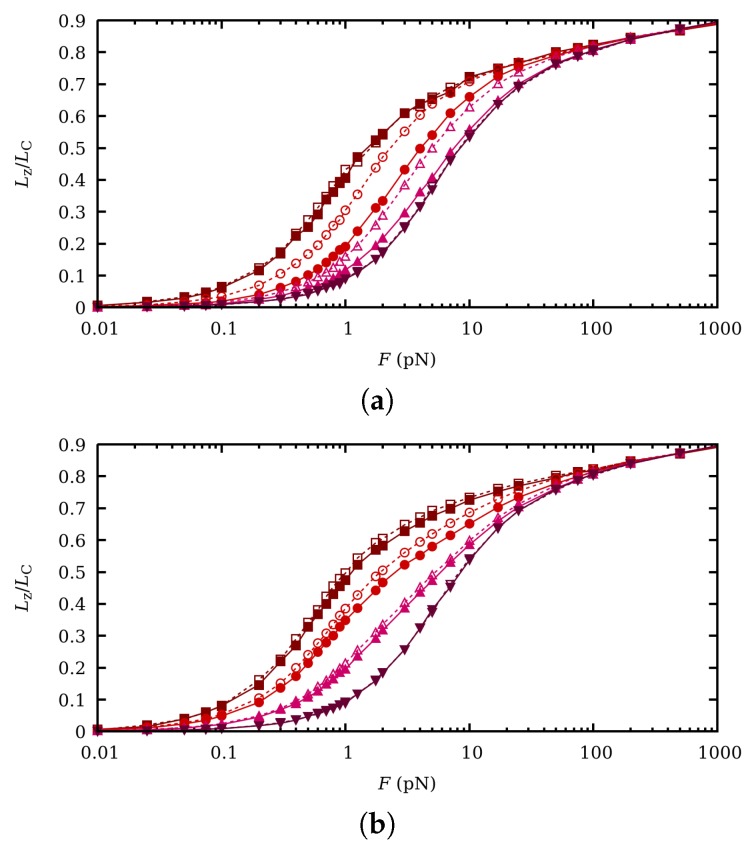
Normalized chain extension versus force curves at fixed pH-values 4 (wine squares), 6 (red circles), 8 (uppwards pink triangles) and 10 (downwards purple triangles), from top to bottom, and ionic strengths 1 M (**a**) and 0.001 M (**b**). Filled markers correspond to SGCMC while empty markers refer to ccMC. The chain extension $L_{z}$ is normalized to the contour length $L_{C}=Nl_{0}\cos\left(\left(\pi -\alpha_{0}\right)/2\right)$.

**Figure 8:**
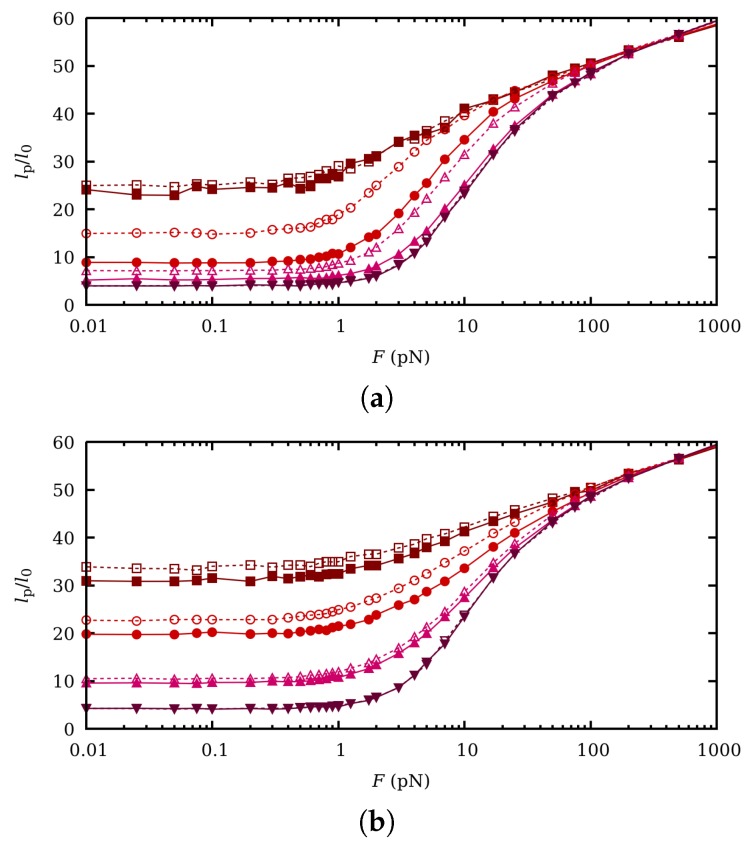
Normalized persistence length $l_{P}$ versus force at pH-values (from top to bottom) 4 (wine squares), 6 (red circles), 8 (uppwards pink triangles) and 10 (downwards purple triangles) and ionic strengths 1 M (**a**) and 0.001 M (**b**). Filled markers correspond to SGCMC while empty markers refer to ccMC. $l_{P}$ is normalized to the equilibrium bond length $l_{0}=1.5$ Å.

The influence of CR and CF in the extension-force curves is evaluated by again comparing the results obtained by SGCMC and ccMC. They are shown in Figure 7 for the same pH-values and ionic strengths as in Figure 6. It is observed that for I=1 M clear differences can be appreciated for pH=8 but specially for pH=6. It is interesting to note that those are the conditions under which the binding capacitance and thus the binding fluctuations are larger (see Figure 2b). As expected, no effect is observed when the polymer is fully charged (pH=4) or fully uncharged (pH=10). For I=0.001 M fluctuations are weaker and the observed differences are smaller than for I=1 M. Although not shown in the figure, we find that SEV does not affect significantly the extension/force curves. Similar trends are found in the persistence length, depicted in Figure 8. Note that, again, the persistence length is a function of the pH-value for intermediate forces due to the activation of the rotational degrees of freedom. The main differences between SGCMC and ccMC also take place for pH=6 and I=1 M, when correlation effects are larger.

## 6. Conclusions

This work addresses the role of charge regulation (CR) and the resulting fluctuations in the conformational and mechanical properties of flexible weak polyelectrolytes, moreover it is motivated by recent findings which suggest that charge regulation can be induced by mechanical stretching [36]. Due to CR, changes in the pH-value modify the average macromolecular charge and conformational equilibria. A second effect is that, for a given average charge per site, fluctuations can alter the intensity of the interactions by means of the correlation between binding sites. We investigate both effects by means of Semi-Grand Canonical Monte Carlo (SGCMC) simulations at constant pH-value, so that the charge is a fluctuating quantity. Once the average charge *per* site is available, we turn off the fluctuations by assigning the same average charge to every site. The molecule is now in a “frozen” ionization state and a MC simulation at constant charge (ccMC) is performed. The main conformational and stretching properties with and without CR are then compared.

We make use of a model which accounts for the main fundamental aspects of a linear flexible polyelectrolyte that is, proton binding, dihedral angle rotation, bond stretching and bending. Steric excluded volume and specific treatment of short range and long-range interactions are also included in the model. This model can be regarded as a kind of “minimal” in the sense that it contains a minimum number of parameters but it still preserves the atomistic detail. We firstly study the case when no external force is applied. It is shown that, if fluctuations are activated, *gauche* state probabilities become larger, and the persistence length smaller, so that the polymer becomes more folded. Electrostatic repulsion is thus enhanced if the charge is fixed and weakened when charge fluctuations, which are quantified by means of the binding capacitance, are present.

In the presence of an applied force, macromolecular stretching is also analyzed with CR (the charge depends on the applied force) and without CR (the charge is fixed to the value at zero force). The analysis of the scaling behavior at the low force regime concludes that Pincus exponent becomes pH-dependent. Both with and without CR, a transition from 1/2 at high pH-values (phantom chain) to 3/5 to low pH-values (Pincus regime), is observed. This fact suggests that Pincus regime is essentially driven by the average charge and that CR plays a minor role. Finally, the intermediate force stretching regime is investigated. It is found that CR induces a moderate influence in the force-extension curves and in the persistence length (which in this force regime becomes force-dependent). It is thus concluded that the effect of CR on the stretching curves is mainly due to changes in the average charge at zero force, although some differences arise at intermediate pH-values. It is also found that the effect of steric excluded volume is almost irrelevant compared to electrostatic self-avoiding interactions for all the cases studied.

## Supplementary Materials

The following are available online at https://www.mdpi.com/2073-4360/11/12/1962/s1, Figure S1: Outline of the metropolis algorithm of the simulation code, Figure S2: Normalized chain extension versus force curves in the low force regime for pH-values ranging from 2 to 10 obtained from ccMC and SGCMC simulations with SEV.

## Abbreviations

The following abbreviations are used in this manuscript:

ccMC	constant charge Monte Carlo
CF	Charge Fluctuation
CR	Charge Regulation
LPEI	Linear PolyEthylenImine
LR	Long Range
MC	Monte Carlo
RIS	Rotational Isomeric State
SB	Site Binding
SBRIS	Site Binding Rotational Isomeric State
SEV	Steric Excluded Volume
SGCMC	Semi-Grand Canonical Monte Carlo
SR	Short Range

## References

[1] Borkovec M., Jönsson B., Koper G.J.M.J., Matigeric E. (2001). Surface and Colloid Science.

[2] Lund M., Jo B. (2013). Charge regulation in biomolecular solution. Q. Rev. Biophys..

[3] Trefalt G., Behrens S.H., Borkovec M. (2016). Charge Regulation in the Electrical Double Layer: Ion Adsorption and Surface Interactions. Langmuir.

[4] Hanakam F., Günther G., Lotz S., Alt T., Seelig A. (1996). Binding of Hisactophilin I and II to Lipid Membranes Is Controlled by a pH-dependent myristoyl–histidine switch. Biochemistry.

[5] Narambuena C.F., Longo G.S., Szleifer I. (2015). Lysozyme adsorption in pH-responsive hydrogel thin-films: The non-trivial role of acid-base equilibrium. Soft Matter.

[6] Lund M., Jönsson B. (2005). On the Charge Regulation of Proteins. Biochemistry.

[7] Mason A.C., Jensen J.H. (2008). Protein-protein binding is often associated with changes in protonation state. Proteins Struct. Funct. Bioinform..

[8] Torres P., Bojanich L., Sanchez-Varretti F., Ramirez-Pastor A.J., Quiroga E., Boeris V., Narambuena C.F. (2017). Protonation of *β*-lactoglobulin in the presence of strong polyelectrolyte chains: A study using Monte Carlo simulation. Colloids Surf. B Biointerfaces.

[9] Torres P.B., Quiroga E., Ramirez-Pastor A.J., Boeris V., Narambuena C.F. (2019). Interaction between *β*-Lactoglobuline and Weak Polyelectrolyte Chains: A Study Using Monte Carlo Simulation. J. Phys. Chem. B.

[10] Carnal F., Clavier A., Stoll S. (2016). Polypeptide-nanoparticle interactions and corona formation investigated by monte carlo simulations. Polymers.

[11] Hamacek J., Borkovec M., Piguet C. (2006). Simple thermodynamics for unravelling sophisticated self-assembly processes. Dalton Trans..

[12] Li Y., Zhao T., Wang C., Lin Z., Huang G., Sumer B.D., Gao J. (2016). Molecular basis of cooperativity in pH-triggered supramolecular self-assembly. Nat. Commun..

[13] Cera E.D. (1991). Stochastic linkage: Effect of random fluctuations on a twostate process Stochastic linkage: Effect of random fluctuations on a two-state process. J. Phys. Chem..

[14] Svensson B., Jonsson B., Thulin E. (1993). Binding of Calcium to Calmodulin and Its Tryptic Fragments: Theory and Experiment. Biochemistry.

[15] Aguilar B., Anandakrishnan R., Ruscio J.Z., Onufriev A.V. (2010). Statistics and physical origins of pK and ionization state changes upon protein-ligand binding. Biophys. J..

[16] Hartig S.M., Greene R.R., Dikov M.M., Prokop A., Davidson J.M. (2007). Multifunctional nanoparticulate polyelectrolyte complexes. Pharm. Res..

[17] Whitten S.T., Garcia-Moreno E.B., Hilser V.J. (2005). Local conformational fluctuations can modulate the coupling between proton binding and global structural transitions in proteins. Proc. Natl. Acad. Sci. USA.

[18] Garcés J.L., Koper G.J.M., Borkovec M. (2006). Ionization Equilibria and Conformational Transitions in Polyprotic Molecules and Polyelectrolytes. J. Phys. Chem. B.

[19] Garcés J.L., Madurga S., Borkovec M. (2014). Coupling of conformational and ionization equilibria in linear poly (ethylenimine): A study based on the site binding/rotational isomeric state. Phys. Chem. Chem. Phys..

[20] Garcés J.L., Madurga S., Rey-Castro C., Mas F. (2017). Dealing with long-range interactions in the determination of polyelectrolyte ionization properties. Extension of the transfer matrix formalism to the full range of ionic strengths. J. Polym. Sci. Part B Polym. Phys..

[21] Blanco P.M., Madurga S., Mas F., Garcés J.L. (2018). Coupling of charge regulation and conformational equilibria in linearweak polyelectrolytes: Treatment of long-range interactions via effective short-ranged and pH-dependent interaction parameters. Polymers.

[22] Giannotti M.I., Vancso G.J. (2007). Interrogation of single synthetic polymer chains and polysaccharides by AFM-based force spectroscopy. ChemPhysChem.

[23] Rief M., Gautel M., Oesterhelt F., Fernandez J.M., Gaub H.E. (1997). Immunoglobulin Domains by AFM Reversible Unfolding of Individual Titin Immunoglobulin Domains by AFM. Science.

[24] Valiaev A., Lim D.W., Oas T.G., Chilkoti A., Zauscher S. (2007). Force-Induced Prolyl Cis - Trans Isomerization in Elastin-like Polypeptides. J. Am. Chem. Soc..

[25] Liu Y., Liu K., Wang Z., Zhang X. (2011). Host-enhanced *π*-*π* Interaction for water-soluble supramolecular polymerization. Chem. Eur. J..

[26] Wang J., Kouznetsova T.B., Niu Z., Ong M.T., Klukovich H.M., Rheingold A.L., Martinez T.J., Craig S.L. (2015). Inducing and quantifying forbidden reactivity with single-molecule polymer mechanochemistry. Nat. Chem..

[27] Radiom M., Kong P., Maroni P., Schäfer M., Kilbinger A.F.M., Borkovec M. (2016). Mechanically induced cis-to-trans isomerization of carbon-carbon double bonds using atomic force microscopy. Phys. Chem. Chem. Phys..

[28] Jacobson D.R., Mcintosh D.B., Stevens M.J., Rubinstein M., Saleh O.A. (2017). Single-stranded nucleic acid elasticity arises from internal electrostatic tension. Proc. Natl. Acad. Sci. USA.

[29] Radiom M., Borkovec M. (2017). Influence of ligand-receptor interactions on force-extension behavior within the freely jointed chain model. Phys. Rev. E.

[30] Stevens M.J., Saleh O.A. (2012). Simulations of Stretching a Strong, Flexible Polyelectrolyte. Macromolecules.

[31] Jacobson D.R., Mcintosh D.B., Saleh O.A. (2013). The Snakelike Chain Character of Unstructured RNA. Biophys. J..

[32] Stevens M.J., Berezneya J.P., Saleh O.A. (2018). The Effect of Chain Stiffness and Salt on the Elastic Response of a Polyelectrolyte. J. Chem. Phys..

[33] Saleh O.A., McIntosh D.B., Pincus P., Ribeck N. (2009). Nonlinear low-force elasticity of single-stranded DNA molecules. Phys. Rev. Lett..

[34] Mcintosh D.B., Saleh O.A. (2011). Salt Species-Dependent Electrostatic Effects on ssDNA Elasticity. Macromolecules.

[35] Stevens M.J., Saleh O.A. (2013). Simulations of Stretching a Strong, Flexible Polyelectrolyte: Using Long Chains To Access the Pincus Scaling Regime. Macromolecules.

[36] Blanco P.M., Madurga S., Mas F., Garcés J.L. (2019). Effect of Charge Regulation and Conformational Equilibria in the Stretching Properties of Weak Polyelectrolytes. Macromolecules.

[37] Kirkwood J.G., Shumaker J.B. (1952). Forces between Protein Molecules in Solution Arising from Fluctuations in Proton Charge and Configuration. Proc. Natl. Acad. Sci. USA.

[38] Odijk T. (1977). Polyelectrolytes Near the Rod Limit. J. Polym. Sci. Polym. Phys. Ed..

[39] Barrat J.L., Joanny J.F. (1993). Persistence Length of Polyelectrolyte Chains. Europhys. Lett..

[40] Ullner M., Jonsson B. (1996). Monte Carlo study of titrating polyelectrolytes in the presence of salt. Macromolecules.

[41] Ullner M. (2003). Comments on the Scaling Behavior of Flexible Polyelectrolytes within the Debye-Hückel Approximation. J. Phys. Chem. B.

[42] Ulrich S., Seijo M., Stoll S. (2007). A Monte Carlo study of weak polyampholytes: Stiffness and primary structure influences on titration curves and chain conformations. J. Phys. Chem. B.

[43] Uyaver S., Seidel C. (2003). First-order conformational transition of annealed polyelectrolytes in a poor solvent. Europhys. Lett..

[44] Flory P. (1967). Statistical Mechanics of Chain Molecules.

[45] Sliozberg Y.R., Kröger M., Chantawansri T.L. (2016). Fast equilibration protocol for million atom systems of highly entangled linear polyethylene chains Fast equilibration protocol for million atom systems of highly entangled linear polyethylene chains. J. Chem. Phys..

[46] Burak Y., Netz R.R. (2004). Charge Regulation of Interacting Weak Polyelectrolytes. J. Phys. Chem. B.

[47] Livadaru L., Netz R.R., Kreuzer H.J. (2003). Stretching response of discrete semiflexible polymers. Macromolecules.

[48] Saleh O.A. (2015). Perspective: Single polymer mechanics across the force regimes. J. Phys. Chem..

[49] Pincus P. (1976). Excluded Volume Effects and Stretched Polymer Chains. Macromolecules.

