# Supplementary material for: Role of Charge Regulation and Fluctuations in the Conformational and Mechanical Properties of Weak Flexible Polyelectrolytes
Source: Polymers (Basel). 2019 Nov 29;11(12):1962. doi: 10.3390/polym11121962 (PMC6960815; doi:10.3390/polym11121962)
Supplement: Supplementary file 1 [file polymers-11-01962-s001.pdf]

polymers

Supplementary materials:

Role of Charge Regulation and Fluctuations in the Conformational and Mechanical Properties of Weak Flexible Polyelectrolytes

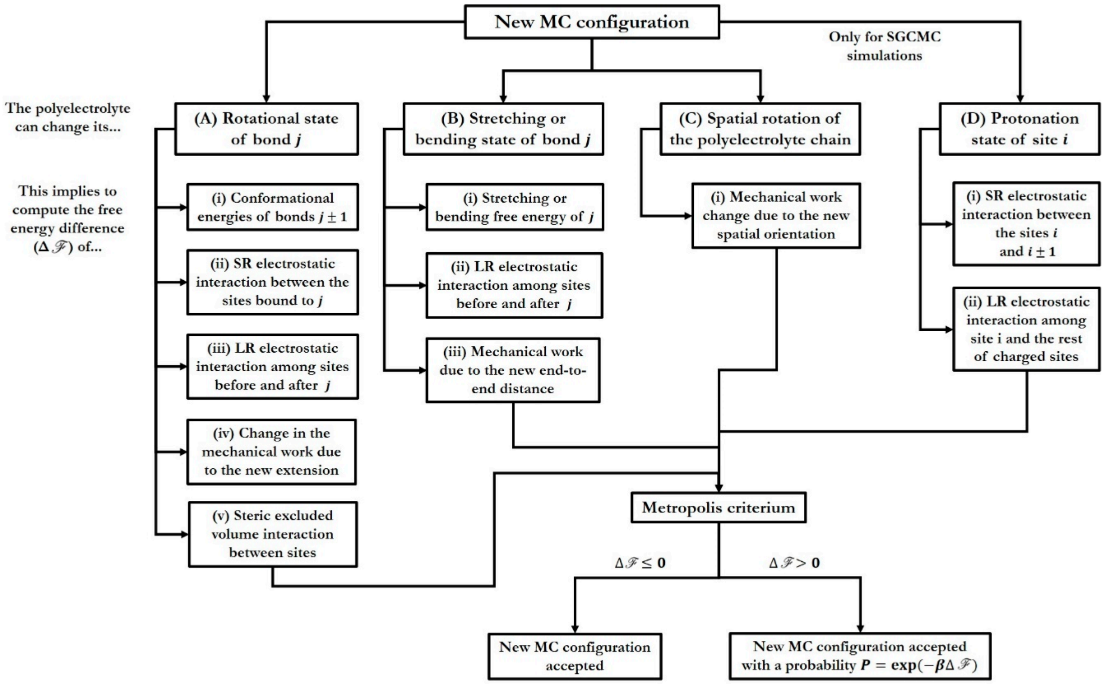

Figure S1. Outline of the metropolis algorithm of the simulation code. In each new Monte Carlo (MC) configuration, the polyelectrolyte can change either (**A**) the rotational state of a bond, (**B**) the length or angle of a bond or (**C**) the spatial orientation of the polyelectrolyte chain in laboratory coordinate frame. In the case of Semi-Grand Canonical Monte Carlo (SGCMC) simulations, the polyelectrolyte can also alter the ionization state of a binding site (**D**).

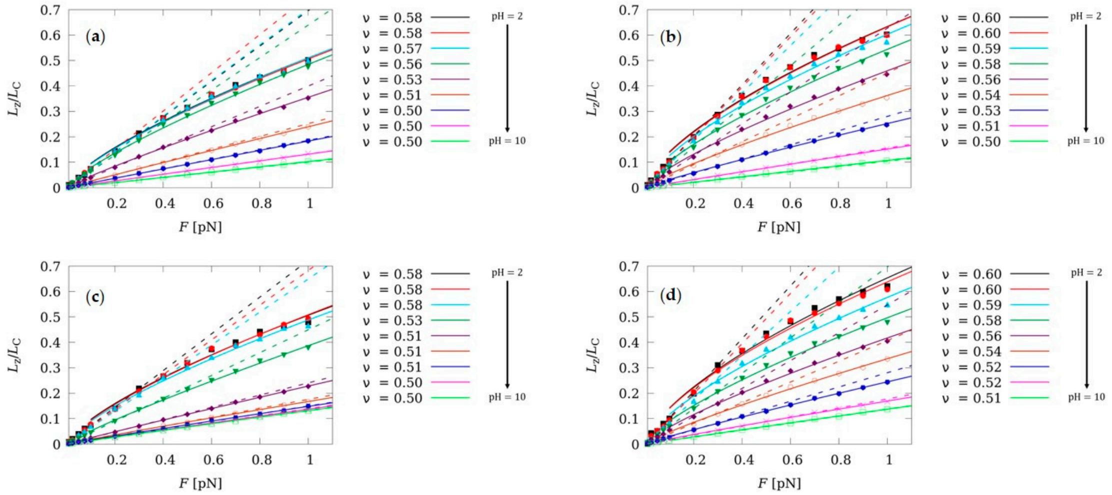

Figure S2. Force F vs. extension L_z curves in the low force regime obtained at different pH-values of 2 (black squares), 3 (red circles), 4 (cyan uppwards triangles), 5 (dark green downwards triangles), 6 (purple diamonds), 7 (ochre empty pentagons), 8 (blue filled pentagons), 9 (pink crosses) and 10 (light green empty squares). The markers show the ccMC (**a** and **b**) and the SMCMC with excluded volume (**c** and **b**) results at two different ionic strengths of 1 M (left) and 0.001 M (right). The dashed lines follow the force/extension linear prediction (Equation 17) using the Kuhn length obtained from simulations at $F = 0$, whereas the continuous ones show the best fit of the computational data to the Pincus scaling low. L_z is normalized to the polyelectrolyte contour length $L_C = Nl_0 \cos((\pi - \alpha_0)/2)$.
